# Neural Mechanisms of Interference Control in Working Memory: Effects of Interference Expectancy and Fluid Intelligence

**DOI:** 10.1371/journal.pone.0012861

**Published:** 2010-09-20

**Authors:** Gregory C. Burgess, Todd S. Braver

**Affiliations:** 1 Institute of Cognitive Science, University of Colorado at Boulder, Boulder, Colorado, United States of America; 2 Department of Psychology, Washington University in St. Louis, Saint Louis, Missouri, United States of America; University College London, United Kingdom

## Abstract

**Background:**

A critical aspect of executive control is the ability to limit the adverse effects of interference. Previous studies have shown activation of left ventrolateral prefrontal cortex after the onset of interference, suggesting that interference may be resolved in a reactive manner. However, we suggest that interference control may also operate in a proactive manner to prevent effects of interference. The current study investigated the temporal dynamics of interference control by varying two factors – interference expectancy and fluid intelligence (gF) – that could influence whether interference control operates proactively versus reactively.

**Methodology/Principal Findings:**

A modified version of the recent negatives task was utilized. Interference expectancy was manipulated across task blocks by changing the proportion of recent negative (interference) trials versus recent positive (facilitation) trials. Furthermore, we explored whether gF affected the tendency to utilize specific interference control mechanisms. When interference expectancy was low, activity in lateral prefrontal cortex replicated prior results showing a reactive control pattern (i.e., interference-sensitivity during probe period). In contrast, when interference expectancy was high, bilateral prefrontal cortex activation was more indicative of proactive control mechanisms (interference-related effects prior to the probe period). Additional results suggested that the proactive control pattern was more evident in high gF individuals, whereas the reactive control pattern was more evident in low gF individuals.

**Conclusions/Significance:**

The results suggest the presence of two neural mechanisms of interference control, with the differential expression of these mechanisms modulated by both experimental (e.g., expectancy effects) and individual difference (e.g., gF) factors.

## Introduction

There has been a long-standing appreciation of the close relationship between working memory (WM) and executive control. In the classic Baddeley model [1], control mechanisms are critical for managing updating and transformation processes applied to information stored in short-term storage buffers. More recently, it has been appreciated that interference may play a fundamental role in limiting the capacity of WM [2], thus highlighting the importance of mechanisms that exert control over interference. Indeed, individual differences in interference control are strong predictors of WM capacity [3], and other domain-general cognitive abilities, such as fluid intelligence (gF) [4], [5].

In the last decade, there has been increased attention within the cognitive neuroscience literature towards understanding the neural mechanisms underlying interference control during WM [6]. This work has highlighted the importance of specific forms of interference – such as that due to previously encoded, but currently irrelevant information (sometimes referred to as proactive interference) – and specific brain regions – such as the left inferior prefrontal cortex (PFC). In the current study, we extend this work, by investigating two factors that may influence the neural mechanisms of interference control, but which have received relatively little attention to date: temporal dynamics and individual differences. Specifically, we examine the distinction between early and late-acting forms of interference control, and how these might be potentially impacted by individual differences in WM-relevant variables such as fluid intelligence.

Much of the recent research examining interference control during WM has utilized the “recent probes” task [6]. In this task, participants see a memory set that contains several items to remember, followed by a brief delay period, and then a single probe item. They are instructed to respond whether the probe item was present in the memory set (i.e., positive probe) or was absent (i.e., negative probe). However, on some proportion of trials, the probe was also “recent”, meaning that it had been presented in the memory set on the *prior* trial. The recency of the probe can lead to a high degree of interference on recent negative trials, as evidenced both by an increased false alarm rate and robustly slower response latencies. In neuroimaging studies, recent negative probes consistently yield greater activation within the inferior frontal gyrus (IFG) compared to nonrecent negative probes. Furthermore, damage to left IFG has been shown to increase susceptibility to interference [7], [8]. Although it is clear that left IFG is involved in control over interference in these studies, other regions have been implicated less consistently, such as dorsolateral prefrontal cortex (DLPFC) [9], [10] and frontopolar cortex [11], [12].

Although the recent probes task has been a useful tool, it emphasizes the temporal dynamics of neural mechanisms involved in *reactive* control over WM interference. Elsewhere [13], [14], we have proposed the Dual Mechanisms of Control (DMC) model, which postulates that cognitive control can operate in both a reactive and proactive fashion. Specifically, we suggest that reactive control processes are late-acting, involving the transient detection and resolution of interference after its onset. In contrast, we suggest that proactive control mechanisms are early-acting, involving the anticipation and prevention of interference prior to its occurrence, via active maintenance of goal-related representations in WM. It has been shown that increased activity for recent negative trials occurs specifically after the onset of the probe item [15]. In the parlance of the DMC model, this pattern is suggestive of a late-acting reactive control mechanism, in that left IFG activates subsequent to the onset of interference, assumingly in an effort to resolve its effects.

It is also possible that interference effects in WM might be reduced through proactive control mechanisms, such as active maintenance of task-relevant goal representations during the delay period. Such representations could be used to bias processing towards task-relevant dimensions of the probe (i.e., its match to current memory set items). Numerous influential models of attentional control have postulated that a byproduct of a top-down bias toward task-relevant processing could be a reduction in the degree of task-irrelevant processing [16]–[18]. In particular, representing the memory set as an attentional set or filter could facilitate rapid processing of the probe to determine its positive/negative status, and thus reduce stimulus-triggered (i.e., automatic, bottom-up) processing of recency information. Therefore, top-down bias may serve as an additional, early-acting control mechanism that prevents the effects of interference before they occur. It has been demonstrated that WM tasks can be performed using proactive or reactive control strategies [14], [19]. However, the possibility that proactive control mechanisms operate to prevent interference during WM has yet to be tested.

We have postulated a number of factors that might modulate the tendency to utilize proactive versus reactive control processes [13]. One critical factor is the expectancy of interference. Under situations in which interference is infrequent and unexpected, reactive control mechanisms are predicted to dominate. In contrast, when interference is relatively frequent and can be reasonably anticipated, there may be a greater tendency for proactive control to emerge. Therefore, in the current study, we manipulated interference expectancy across separate conditions. In the low expectancy (LE) condition, only 20% of recent probes were recent negatives, whereas in the high expectancy (HE) condition, 80% of recent probes were recent negatives. Recency and interference were de-confounded by holding probe recency constant at 50% overall in both blocks (by complementing the recent negative probes with recent positives: 80% in LE; 20% in HE). Thus, we predicted that the selective association between recency and interference in the HE condition would lead to a greater utilization of proactive control, which should be reflected as increased delay-period activation relative to the LE condition. Conversely, in the LE condition, the primary dependence on reactive control should be reflected as increased probe-related activation, especially on recent negative trials, replicating prior results [15].

A secondary goal of the study was to examine the influence of fluid intelligence (gF) on the utilization of proactive versus reactive control over interference during WM. It has been shown that gF is highly related to WM span [20], and it has been suggested that this relationship arises from the ability to actively maintain task-relevant information in the face of interference [21]. However, it has not been shown whether decreased susceptibility to interference affects the ability to maintain information in WM, or if the ability to maintain information in WM leads to decreased susceptibility to interference. We have postulated [13] that individuals with higher levels of gF may show a greater tendency to utilize proactive control mechanisms than low gF individuals.

Prior studies from our lab [5] have shown that high gF individuals are less susceptible to adverse effects of interference during performance of a different WM task – the n-back. However, due to the continuous nature of the n-back task, it was not possible to determine whether more effective interference control was reflected in proactive or reactive mechanisms. An advantage of the recent probes task is the ability to investigate the temporal dynamics of interference control to determine whether they result in early (delay-related, proactive) or late (probe-related, reactive) activation patterns. We predicted that high and low gF individuals would differ in the temporal dynamics of interference control, with interference control for high gF individuals occurring primarily during the delay period, and interference control for low gF individuals occurring primarily during the probe period.

## Materials and Methods

### Subjects

Twenty-two participants were recruited from Washington University, St. Louis and the surrounding community to participate in this experiment. All fMRI participants were right-handed, native English speakers, and screened to ensure no neurological or psychiatric disorders, psychotropic medications, or other factors contraindicating fMRI. The research protocol was approved by the institutional review board of Washington University, St. Louis (Human Research Protection Office), and all participants provided written informed consent prior to participation.

The participants were recruited to participate in the fMRI session based on their performance in a prior behavioral session in which the Raven's Advanced Progressive Matrices (RAPM) [22] – a widely utilized gF measure – was assessed. The participants recruited for the current fMRI study had scored either in the upper or lower quartile of a sample that included an additional 38 participants. Three participants were excluded from analyses due to technical problems (2 participants) or excessive head movement (1 participant). Thus, results are reported from the remaining 19 participants (10 male, age range 18–35). Ten individuals were in the high gF group (*mean RAPM*  = 30.40 out of 36, *SD*  = 1.50) and nine were in the low gF group (*mean RAPM*  = 21.00 out of 36, *SD*  = 2.06). As such, the study can be considered an extreme-groups design.

### Task

Participants were scanned while performing a modified recent negatives paradigm, involving a 5-item memory set. The stimuli were English words, all one- or two-syllable nouns, between 4–6 letters in length. Each trial consisted of the following series of events: memory set presentation (2.5 sec), delay interval (5 sec), probe period (2 sec). Probe responses were button presses indicating whether the probe word was an item in the immediately presented memory set (*positive probe*; right index finger) or was not a member of this set (*negative probe*; right middle finger). Positive and negative probes were randomly intermixed with equal frequency in all blocks (i.e., 50% positive, 50% negative probes). Probe recency (i.e., whether the probe was included in a previous trial's memory set) was also manipulated on a trial-by-trial basis, with 50% recent and 50% novel probes. However, across blocks, recency was differentially associated with interference by varying the frequency of recent negative vs. recent positive probes. Within the LE condition, 10% of trials were recent negatives, 40% were recent positives, 40% were novel negatives, and 10% were novel positives. Within the HE condition, 40% of trials were recent negatives, 10% were recent positives, 10% were novel negatives, and 40% were novel positives.

Interference expectancy conditions (LE, HE) were performed in a blocked fashion in separate scanning runs. Ten scan runs were acquired, with five blocks of one interference expectancy condition, followed by five blocks of the other interference expectancy condition, with the order of interference expectancy conditions counterbalanced across subjects. Each run lasted 412.5 seconds, and consisted of 2 blocks of trials (150 seconds each), alternating with 3 fixation blocks (37.5 seconds each). Each block of trials consisted of 10 trials intermixed with 20 null fixations, for a total of 100 trials per expectancy condition. Visual stimuli were presented using PsyScope software [23] running on Apple PowerMac G4. Stimuli were projected to participants with an LCD projector onto a screen positioned at the head end of the bore. Participants viewed the screen through a mirror attached to the head coil. A fiber-optic, light-sensitive key press interfaced with the PsyScope Button Box was used to record participants' behavioral performance.

### fMRI acquisition and analyses

Whole-brain images were collected on a Siemens 1.5 Tesla Vision System (Erlangen, Germany) with a standard circularly polarized head coil. High-resolution (1.25×1×1) structural images were acquired using a sagittal MP-RAGE 3D T1-weighted sequence. Functional images were acquired using an asymmetric spin-echo, echo-planar sequence (TR  = 2500 ms, TE  = 50 ms, flip angle  = 90°) that was sensitive to blood-oxygen-level-dependent (BOLD) magnetic susceptibility. Each of the 10 scanning runs provided 165 whole-brain volumes consisting of 16 contiguous, 8-mm thick axial slices, acquired parallel to the anterior-posterior commissure plane (3.75×3.75 mm in-plane resolution). Functional images were movement and artifact corrected, intensity normalized within each scanning run, and temporally aligned within each brain volume. Prior to statistical analyses, functional images were re-sampled into 3 mm isotropic voxels, transformed into atlas space, and smoothed with a 9 mm FWHM Gaussian filter. During task blocks, the inter-trial interval varied from 1 TR to 4 TRs, in an optimized logarithmic distribution, in order to create the necessary temporal jitter to allow deconvolution of event-related fMRI responses. Four null volumes were included in each scanning run to allow the scanner to ensure equilibrium of longitudinal magnetization, and were discarded prior to analysis.

A general linear model approach [24] was used to estimate parameter values for event-related responses. Event-related effects were analyzed by computing parameter estimates for each time point within the hemodynamic response epoch (i.e., 10 delta-function regressors, one for each of the 10 TRs; total 25s). This approach to GLM estimation (as opposed to a fit to predefined hemodynamic response function model) has been found to be critical in estimating complex trials or multi-event-related responses in rapid event-related designs. Separate regressors were included for various nuisance effects (e.g., linear drift), such that the parameter estimates were statistically free of influence of those effects. The mean GLM estimates during the 4^th^ and 5^th^ time points (7.5s to 12.5s) were chosen to operationally define delay-related activity, while the mean estimates during 7^th^ and 8^th^ time points (15s to 20s) were chosen to operationally define probe-related activity.

The delay-related activity corresponds to the two TRs subsequent to the initial peak of the HRF in V1. Selecting time points subsequent to the peak in V1 increased the likelihood that positive deflections in activation reflect processes that occurred after the memory set (i.e., during the delay period). These processes may include, but are not limited to, the active maintenance of goal representations and task-relevant information. However, activity from these time points may also contain residual or extended processing from the memory set period, such as increased encoding or depth of processing of the memory set items.

Probe-related activity corresponds to the two TRs that occur during the peak in primary motor cortex (7^th^ and 8^th^ time point). Therefore, interference-control processes that affect activation at these time points likely occur concurrent with response selection and commission. Importantly, differences between recent and novel probes at these time points are unlikely to reflect activation prior to the onset of the probe, because the identity of the probe as recent or novel is unknown until its presentation.

An *a priori* ROI-based approach was used to identify regions showing interference effects during WM. Analyses were restricted to twenty-five ROIs within the canonical network engaged by WM and executive control tasks, as defined by previous meta-analyses (See the second table in [25] and the fourth table and part B of the fifth figure in [26].) and a review that focused on cognitive control over memory (See the average stereotaxic coordinates in the second table in [27].). The resulting mask image, generated from spherical ROIs (10 mm radius) centered on these published anatomical coordinates (Table 1), contained 3541 voxels, or 95,607 mm^3^ in volume.

**Table 1:** Centers of mass used to create a priori ROI mask for neuroimaging analyses.

Study	Region	X	Y	Z
Owen et al. (2005)				
	Lateral premotor (BA 6)	28	0	52
		−26	2	52
	Dorsal cingulate/SMA (BA 32/6)	−2	12	42
	DLPFC (BA 9/46)	42	32	30
	VLPFC (BA44)	−50	12	8
		−62	0	14
	Frontal pole (BA 10)	−38	44	20
		36	46	18
	Medial posterior parietal (BA 7)	12	−64	48
	Inferior parietal lobe (BA 40)	30	−58	42
		38	−46	38
		−34	−48	38
Wager and Smith (2003)				
	BA 10, 9, 46, 47	−32	44	22
	BA 9, 6	−45	7	32
	BA 40, 39, 7	−37	−51	41
	BA 9, 10, 46	36	36	28
	BA 7, 40	31	−59	43
	BA 47, 10, 11, 13	34	31	−4
	BA 7	−12	−70	46
	BA 6, 32, 8	0	11	49
	BA 6	27	0	56
	BA 6, 9, 44	45	1	29
	BA 6	−28	−4	56
Badre and Wagner (2007)				
	Anterior VLPFC	−48	30	−6
	Mid-VLPFC	−50	25	14

BA: Brodmann Area; SMA: supplementary motor cortex; DLPFC: dorsolateral prefrontal cortex; VLPFC: ventrolateral prefrontal cortex.

This ROI mask was used to constrain analysis to only those voxels that were theoretically expected to be strongly associated with interference control during WM. We then identified voxel clusters from within these masks that showed particular interference control effects of interest. These effects were tested through multiple contrasts, and a voxel cluster was only identified if it simultaneously satisfied each of the contrasts. For reactive control ROIs, we identified voxels that showed significantly greater activity for recent negative than novel negative probes, and also activity for recent negative probes was significantly greater than fixation baseline. For proactive control ROIs, we identified voxels in which delay activity during HE trials was greater than fixation baseline, and also that delay activity was greater for HE trials than LE trials. To correct for multiple comparisons, we conducted Monte Carlo simulations as implemented in AlphaSim [28]. This procedure found that contiguous clusters including seven or more voxels (> = 189 mm^3^) showing two significant effects each at p<.025 were corrected for multiple comparisons at an alpha level of .05.

## Results

### Behavioral Results

Previous studies utilizing the recent probes paradigm have shown behavioral interference effects (decreased accuracy and slower RTs) for recent negatives relative to novel negatives. Furthermore, some studies have also shown facilitation (greater accuracy and faster RTs) for recent positives relative to novel positives. We examined both interference and facilitation effects due to recency, as well as whether these effects were modulated by gF and interference expectancy.

First, we conducted an ANOVA on error rates for negative probes with gF, interference expectancy, and recency as factors (Table 2). We identified a robust recency effect, F(1,17)  = 25.3, p<.001, which arose from an 11.8% interference effect for recent negatives compared to novel negatives. There was also a main effect of gF, F(1,17)  = 6.9, p = .018, with fewer errors for the high gF group (3.3%) compared to the low gF group (9.7%). More importantly, these two factors interacted, such that low gF individuals showed a more pronounced interference effect than high gF, F(1,17) = 5.3, p = .035 (low g = 17.5%; high gF = 6.7%). There were no significant effects of, or interactions with, interference expectancy.

**Table 2:** Behavioral performance during recent probes task.

		Negative Probes		Positive Probes	
		Novel	Recent	Novel	Recent
High gF	HE	100%	95%	96%	94%
		(852ms)	(1056ms)	(852ms)	(923ms)
	LE	100%	92%	93%	95%
		(912ms)	(1135ms)	(903ms)	(955ms)
Low gF	HE	99%	81%	93%	90%
		(916ms)	(1145ms)	(936ms)	(977ms)
	LE	99%	82%	91%	94%
		(880ms)	(1102ms)	(918ms)	(950ms)

Average accuracy and RT (in parentheses) for responses during the recent negatives task. HE: high interference expectancy; LE: low interference expectancy; gF: fluid intelligence.

Second, we tested a similar ANOVA on RT to determine whether gF, interference expectancy, and recency affected response latency for negative probes (Table 2). Again, a robust recency effect was observed, F(1,17) = 76.4, p<.001, with significant interference effect observed for recent negative probes (1109 ms) compared to novel negative probes (890 ms). However, no other main effects or interactions were significant, suggesting that the magnitude of this interference effect was not modulated by gF or interference expectancy.

Because some previous studies have found recency effects for positive probes (i.e., response facilitation) as well as negative probes [11], we also conducted a separate analysis on these trials (Table 2). For accuracy, there were no significant facilitation effects for positive probes due to recency, or interactions with gF and interference expectancy (all p values >.07). There was a recency effect in terms of RT for positive probes, F(1,17) = 10.9, p = .004. However, this effect resulted from *slower* RT for recent positive probes relative to novel positive probes, rather than facilitation. There were no other significant effects or interactions due to gF or interference expectancy (all p values >.25).

Although effects of interference expectancy on behavioral performance were not statistically significant in the current sample, it should be noted that evidence was present in a prior pilot sample suggesting that the expectancy manipulation can affect interference control performance. Data from a pilot sample of 41 participants suggested that interference expectancy affected the degree to which recency information influenced responding. Specifically, in that study, there was a significant expectancy x recency x target interaction on RT, F(1,40) = 5.162, p = .029. RT interference for negative probes tended to be greater in the LE condition than in the HE condition (187 ms vs. 145 ms), although the expectancy difference was not significant, F(1,40) = 2.288, p = .138. Similarly, facilitation for positive probes was present in the LE condition (30 ms), but not in the HE condition (−11 ms); that expectancy difference was statistically significant, F(1,40) = 4.581, p = .038. Also in the pilot sample, the high gF group (based on median split) demonstrated the 3-way interaction between expectancy, recency and target status seen in the full group data, F(1,19) = 8.015, p = .011, with the HE condition showing both significantly reduced interference for negative probes (201 msec vs. 114 msec; F(1,19) = 4.586, p = .045) and significantly reduced facilitation for positive probes (36 msec vs. –26 msec; F(1,19) = 5.941, p = .025). However, within the low gF group, the expectancy x recency x target interaction was not present, F(1, 20) = 0.181, p = 0.675, nor were the specific expectancy effects on negative probe interference or positive probe facilitation (both F's<1). Therefore, although we believe that expectancy condition can affect interference control, the behavioral effects are relatively small and likely interact with gF. Consequently, it is possible that the effect of expectancy was not statistically significant in the imaging sample simply due to a lack of power related to this small effect size.

In summary, the behavioral results replicate the critical finding of interference for recent negative probes relative to novel negative probes. Furthermore, the results also suggest that high gF individuals had fewer errors overall than low gF individuals, and more specifically, a reduced tendency to make errors on recent negative trials. This pattern supports the hypothesis that high gF individuals can exhibit better interference control than low gF individuals.

### Imaging effects

The first step was to identify those brain regions that demonstrated interference-related probe activity, as defined by increased activity on recent negative probes, both relative to fixation and to novel negative probes. Because we hypothesized that interference expectancy might affect the neural mechanisms used to control interference during the task, we conducted separate analyses for the HE and LE conditions.

#### Recency effect for negative probes in LE condition

Six ROIs showed a pattern of interference-related probe activity during the LE condition, located within left DLPFC and lateral parietal lobe, right pre-SMA, as well as bilateral IFG (Table 3). Indeed, the left IFG ROI replicates well the anatomical location associated with recent negative interference in many prior studies. For each subject, interference-related probe activity (i.e., percent signal change for recent negative probe activity versus novel negative probe activity) was extracted separately for the HE and LE conditions from each of the six ROIs. Separate ANOVAs on interference-related probe activity from the six ROIs failed to show effects of gF, interference expectancy, or interactions between gF and interference expectancy for these ROIs (all p's >.05).

**Table 3:** Regions that show interference-related effects during probe or delay periods.

Region	BA	Peak Z	mm^3^	X	Y	Z	% sc
Negative Probe ROIs HE condition							
Middle frontal gyrus (L)	9/44	3.22	2376	−46	8	33	0.16%
Negative Probe ROIs LE condition							
Precentral gyrus (L)	6	2.84	270	−34	−4	51	0.06%
Medial frontal gyrus (R)	6	3.21	1620	1	9	49	0.13%
Inferior parietal lobule (L)	40	2.63	540	−36	−58	41	0.07%
Precentral gyrus (L)	6	2.78	486	−48	7	36	0.12%
Inferior frontal gyrus (L)	44/45	3.24	1377	−52	13	8	0.20%
Inferior frontal gyrus/insula (R)	47/13	2.60	297	39	24	0	0.14%
Positive Probe ROIs LE condition							
Middle frontal gyrus (L)	10/46	2.67	351	−37	48	12	0.13%
Inferior parietal lobule (R)	40	2.28	540	44	−51	39	0.08%
Inferior parietal lobule (L)	40	2.32	243	−40	−57	36	0.11%
Delay ROIs							
Inferior frontal gyrus (L)	47	3.84	513	−41	25	−6	0.44%
Inferior frontal gyrus (R)	47	2.75	189	37	22	−6	0.42%
Middle frontal gyrus (L)	45/46	3.05	1863	−42	29	13	0.35%
Middle frontal gyrus (R)	9	3.34	1971	36	32	25	0.29%
Precentral gyrus (L)	6	3.10	756	−58	0	19	0.40%
Precentral gyrus (R)	6	3.70	3969	44	0	30	0.36%
Middle frontal gyrus (L)	9	3.00	1539	−47	2	31	0.36%
Medial frontal gyrus (R)	6/32	2.73	2619	0	11	48	0.36%
Middle frontal gyrus (L)	6	3.09	1998	−25	−2	48	0.27%
Middle frontal gyrus (R)	6	2.68	1215	27	−3	52	0.24%
Inferior parietal lobule (R)	40	2.32	270	37	−42	46	0.34%

HE: high interference expectancy; LE: low interference expectancy; BA: Brodmann Area; Peak Z: z-statistic for effect at peak voxel; mm^3^: total volume of ROI in cubic millimeters; %sc: percent signal change; %sc for interference-related probe ROIs is difference between recent negative and novel negative activity during probe period; %sc for target recency effect is activation difference for recent positives versus novel positives during probe period; %sc for interference-related delay ROIs is difference between HE delay activity and LE delay activity.

#### Recency effect for negative probes in HE condition

Only one region – left middle frontal gyrus (BA9/44) – demonstrated a pattern of interference-related probe activity during the HE condition (Table 3). The percent signal change for the recency contrast (i.e., recent negative probe activity versus novel negative probe activity) was averaged across all voxels in the ROI, yielding an estimate of interference-related probe activity. This estimate of interference-related probe activity was extracted for each subject separately for the HE and LE conditions. Again, an ANOVA on interference-related probe activity failed to show a significant effect of interference expectancy, gF, or an interaction between gF and interference expectancy for this ROI (all p's >.15).

Previous studies have demonstrated that recency had similar effects on activation of positive probes as is typically observed for negative probes. Therefore, we searched for regions that showed significantly increased activity for recent positive probes, both relative to novel positive probes and fixation. Again, these analyses were conducted separately for the HE and LE conditions, to allow the possibility that interference expectancy might influence the neural mechanisms used to control effects of recency during the task.

#### Recency effect for positive probes in LE condition

Three ROIs, located within left middle frontal gyrus (BA10/46) and bilateral parietal lobe (BA40), showed increased activation for recent positive probes during the LE condition (Table 3). The ROI within left BA10/46 is close to the anatomical location associated with recent positive activation in prior studies [11], [12]. For each subject, the recency effect for positive probes (i.e., percent signal change for recent positive versus novel positive probe activity) was extracted separately for the HE and LE conditions. Separate ANOVAs on probe activity from the three ROIs failed to reach significance for effects of gF, interference expectancy, or interactions between gF and interference expectancy for these ROIs (all p's >.05).

#### Recency effect for positive probes in HE condition

A search for regions demonstrating significantly increased activity during the HE condition for recent positive probes, relative to both novel positive probes and fixation, yielded no significant ROIs.

An additional analysis was conducted to identify brain regions that demonstrated interference-related activity during the delay, rather than probe period. As discussed previously, we hypothesized that greater interference expectancy might engage neural mechanisms of proactive control that act to prevent interference before its onset. Therefore, interference-related effects during the delay were defined by increased delay-period activity in the HE condition, relative to both the fixation baseline and delay-period activity in the LE condition. Because the presence of interference on any particular trial is unknown prior to the probe period, delay activity is not expected to vary with the recency and target status of the probe [15]. Therefore, in these analyses, delay-period activity was averaged across all trial types.

#### Effect of interference expectancy on delay activity

This analysis identified eleven interference-related delay regions. These regions fell within bilateral DLPFC, IFG, and pre-SMA, and within right lateral parietal regions (Table 3). We estimated interference-related delay activity by averaging the percent signal change for the delay contrast (i.e., delay activity averaged across all trial types versus baseline) across all voxels in each ROI, separately for the HE and LE conditions. An ANOVA on delay-related activity from these regions confirmed significantly greater activity during the HE condition (0.90% signal change) versus LE condition (0.55% signal change) F(1,17) = 21.7, p<.001. However, the gF effect and gF x expectancy interaction were not significant.

The above analysis procedure was conducted to detect regions that showed greater activity during the HE condition than the LE condition. To investigate the specificity of these effects, we conducted a parallel analysis to identify any possible regions that showed more delay activity for the LE than HE condition. This parallel analysis found no voxel clusters that showed this opposite effect within the *a priori* regions of interest within the WM/executive control network. This suggests that the pattern of interference-related activity during the delay period was not simply an artifact of the analysis method, but rather a specific effect of interference expectancy on components of the core brain network associated with WM and interference control.

**Figure 1:**
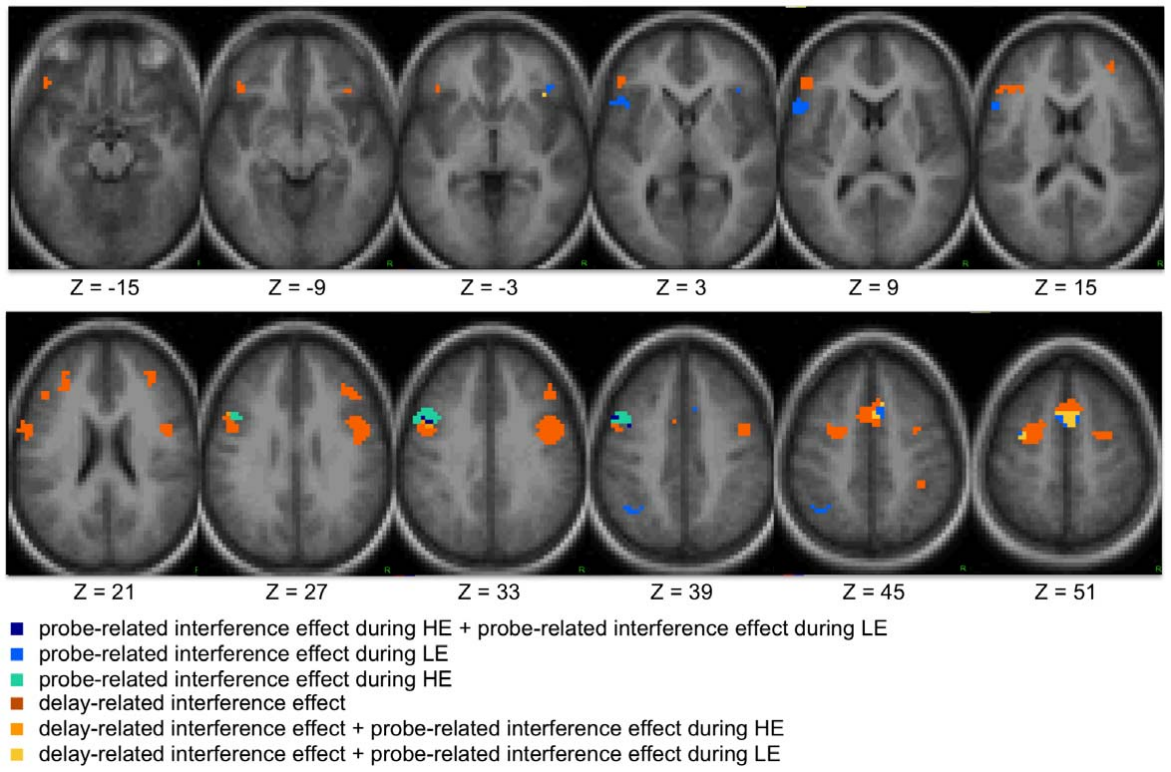
Overlap among ROIs that show interference-related effects during probe and during delay. Voxels are color-coded according to the interference-related effects demonstrated by that region. Only pre-SMA (Z = 45 and Z = 51) shows substantial overlap of interference-related effects during the probe and delay periods. LE: low interference expectancy; HE: high interference expectancy.

**Figure 2:**
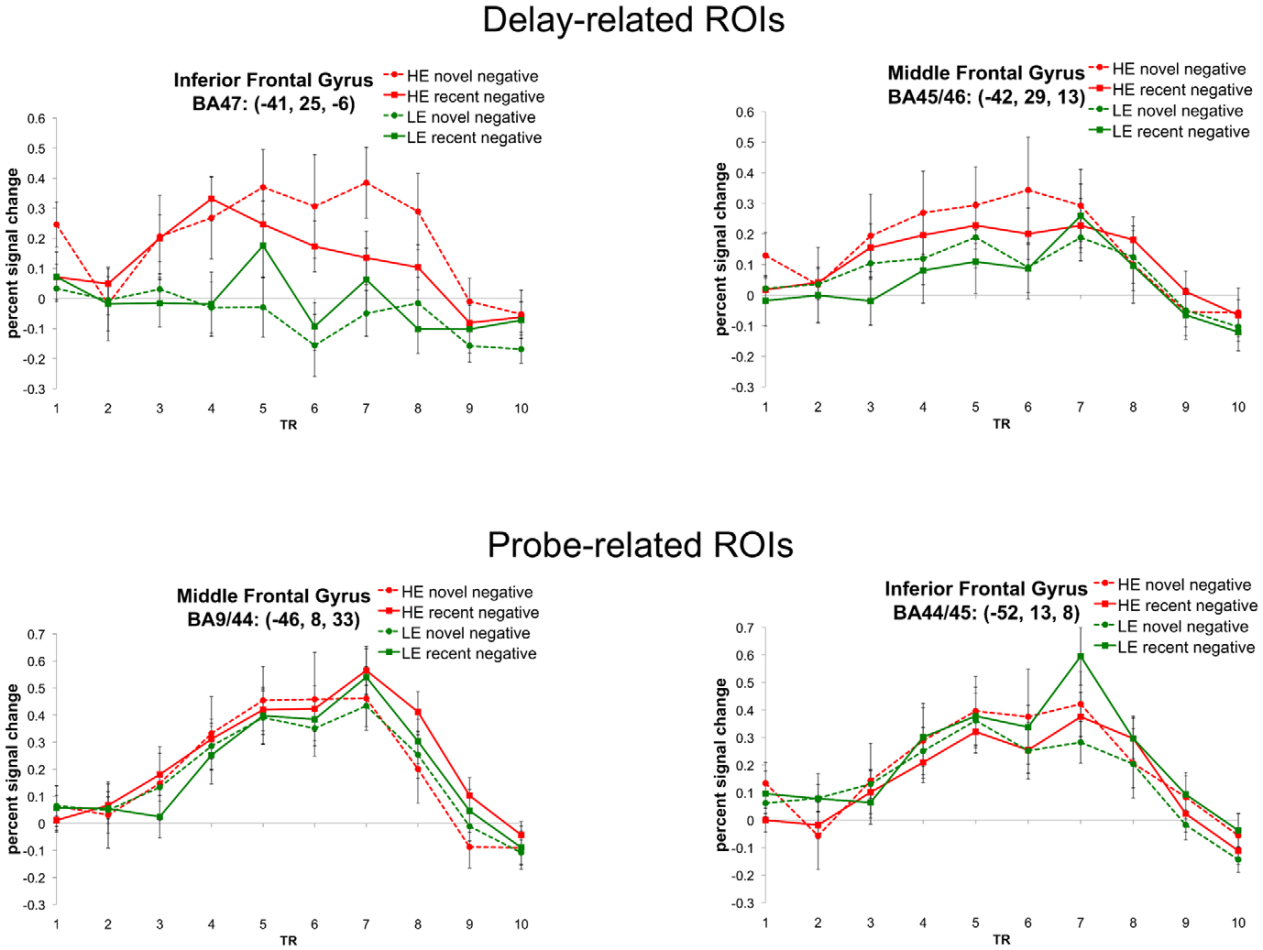
Timecourse plots from interference-related regions within left lateral PFC. Left lateral PFC regions did not show significant interference-related effects during both the delay and probe periods. These four panels show percent signal change for negative trials as a function of time. The top two panels are timecourses from ROIs that showed interference-related effects during the delay period. The bottom two panels are timecourses from ROIs that showed interference-related effects during the probe period. Time points 4 and 5 reflect delay-period activity. Time points 7 and 8 reflect probe-period activity. HE: high interference expectancy; LE: low interference expectancy; BA: Brodmann Area; TR: relaxation time (time point).

The results of recent studies [14] and unpublished data from our laboratory suggest that some PFC regions might be capable of flexibly switching between proactive and reactive control modes. In the current study, several anatomical regions showed effects consistent with both proactive control during the HE condition and reactive control during the LE condition. Therefore, we conducted a formal overlap analysis to investigate the degree to which specific regions exhibited a switch from one control mode to another depending upon interference expectancy (Figure 1). This analysis failed to show regions in left IFG that showed both probe-related and delay-related interference effects. We selected the two delay-related ROIs and two probe-related ROIs that were nearest to the canonical LIFG region in previous studies of probe-related interference effects. Timecourses extracted from the delay-related ROIs did not show significant probe-related effects, and timecourses extracted from the probe-related ROIs did not show significant differences between delay activity during HE and LE conditions (Figure 2).

However, outside of left IFG, the overlap analysis identified two probe-related interference ROIs that overlapped substantially with delay-related interference effects. First, the probe-related interference ROI in left precentral gyrus (premotor cortex; peak coordinate: −34, −4, 51) was adjacent to a larger, more medial region that showed a delay-related interference effect (middle frontal gyrus; peak coordinate: −25, −2, 48). Fifty percent of the probe-related interference ROI (135 mm^3^) overlapped with the delay-related interference ROI. However, as a portion of the total volume of the combined adjacent regions (2133 mm^3^), only 6.3% of the region showed both probe-related and delay-related interference effects. Therefore, the pattern of activation in this portion of lateral PFC may be best characterized by its delay-related effects. Second, a region in right medial frontal gyrus (pre-SMA) showed both probe-related and delay-related interference effects. Within this pre-SMA region, the more anterior portion showed a delay-related interference effect, the more posterior portion showed a probe-related interference effect, and the central portion showed both effects. Of the total volume of the combined interference-related region (3294 mm^3^), 28.7% showed both probe-related and delay-related interference effects. Consequently, this suggests that pre-SMA may be best characterized as showing both forms of interference control.

#### Subregions sensitive to gF differences

The ANOVAs reported above failed to show relationships of gF with interference-related probe activity or interference-related delay activity. However, previous studies from our laboratory have demonstrated activation differences between high gF and low gF groups in interference-control regions [5]. The current behavioral results replicated the findings of reduced interference effects for high gF versus low gF individuals. Our *a priori* prediction was that high and low gF groups would recruit interference-control regions differentially. Although our analyses did not show differential recruitment by the gF groups when averaged across all voxels within interference-control ROIs, it was possible that this null effect reflected reduced power for these analyses (i.e., true gF effects in smaller sub-clusters were diluted by the larger number of voxels that only showed interference-related activity on average). Given our *a priori* hypotheses and behavioral results consistent with those hypotheses, we conducted an exploratory analysis to determine if there were significant effects of gF, or interactions between gF and interference expectancy, within smaller clusters of the ROIs identified above.

**Figure 3:**
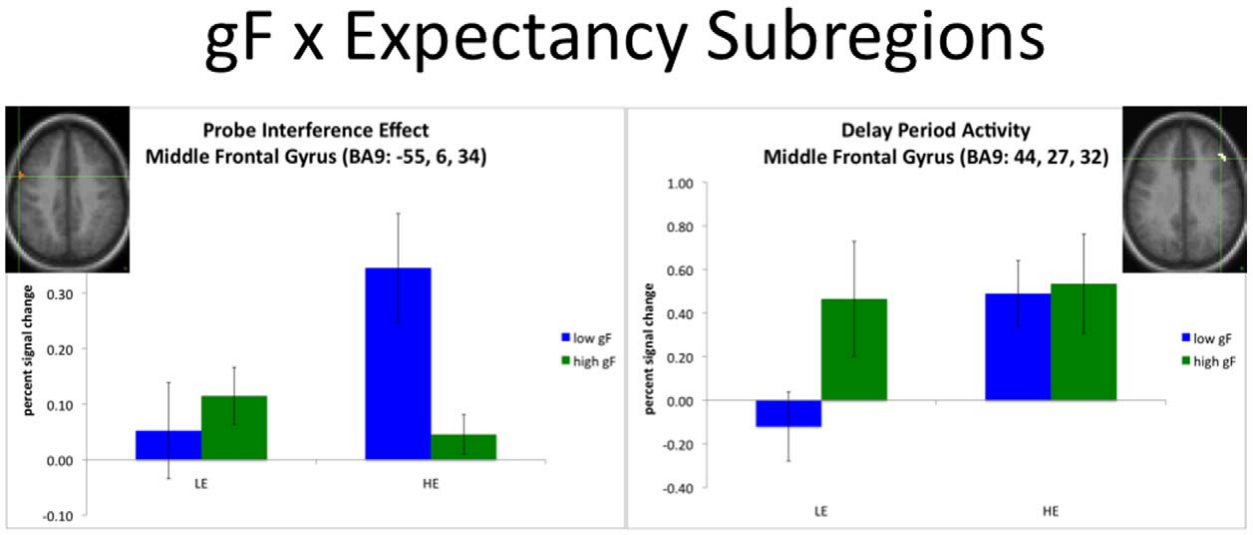
Interference-related effects from subregions that show gF x expectancy interactions. Subregions that showed significant gF x expectancy interactions suggest that high gF individuals control interference through delay-period activation, whereas low gF individuals control interference through probe-period activation. Activity in these subregions is shown separately for high gF and low gF groups during the HE and LE conditions. The figure on the left shows percent signal change for interference-related effects during the probe period (recent negatives – novel negatives) in left middle frontal gyrus. The figure on the right shows percent signal change for activity during the delay period (averaged across all trials) in right middle frontal gyrus.

Within the six ROIs showing interference-related probe activity during the LE condition, there were no subregions that showed significant gF effects or gF x interference expectancy interactions. Similarly, the three ROIs that showed recency effects for positive probes during the LE condition failed to show gF effects or interactions between gF and interference expectancy. However, for the ROI that demonstrated interference-related probe activity in the HE condition, there was a small subregion (left middle frontal gyrus, BA9; Table 4 and Figure 3) that showed a significant gF x interference expectancy effect, F(1,17) = 11.6, p = .003. Interestingly, for this subregion, the gF x interference expectancy interaction arose because the low gF group showed a larger interference effect than the high gF group during the HE condition, F(1,17) = 7.6, p = .014, whereas during the LE condition there was no difference between the two groups, F<1. This pattern of activity is inconsistent with the hypothesis that increased probe-related activity is associated with better interference control, because the low gF group showed increased probe-related activation of this region, but behaviorally showed poorer interference control.

**Table 4:** Subregions of interference-related ROIs that show gF effects or interactions between gF and expectancy.

Region	BA	mm^3^	X	Y	Z	Exp	High gF %sc	Low gF %sc
Probe ROIs HE condition (gF x exp effect subregion)								
Middle frontal gyrus (L)	9	189	−55	6	34	HE	0.04%	0.34%
						LE	0.11%	0.05%
Delay ROIs (gF effect subregion)								
Medial frontal gyrus (R)	6	378	3	16	47	HE	1.09%	0.61%
						LE	0.78%	0.10%
Precentral gyrus (R)	6	216	30	−5	51	HE	1.09%	0.62%
						LE	0.91%	0.34%
Delay ROIs (gF x exp effect subregion)								
Middle frontal gyrus (R)	9	324	44	27	32	HE	0.53%	0.49%
						LE	0.47%	−0.12%

Exp: interference expectancy condition; HE: high interference expectancy; LE: low interference expectancy; BA: Brodmann Area; mm^3^: total volume of ROI in cubic millimeters; gF: fluid intelligence; %sc: percent signal change; %sc for interference-related probe ROIs is difference between recent negative and novel negative activity during probe period; %sc for interference-related delay ROIs is difference between average delay activity and fixation baseline.

A contrasting pattern was found within ROIs that showed sensitivity to interference expectancy during the delay period. Two subregions showed significant effects of gF (right pre-SMA and right posterior PFC; Table 4). In pre-SMA, delay activity was significantly greater, F(1,17) = 6.5, p = .020, for the high gF group than the low gF group collapsing across interference expectancy condition. The right posterior PFC subregion showed a very similar pattern of significantly higher delay-related activity in the high gF compared to low gF group, F(1,17) = 6.4, p = .022. A third sub-cluster was also identified, in right DLPFC (Table 4 and Figure 3), that showed a significant interaction between gF and interference expectancy, F(1,17) = 7.6, p = .010. This interaction arose because there was a trend towards greater delay-related activity in the high gF group during the LE condition, F(1,17) = 2.99, p = 0.10, but not during the HE condition, F<1.

Together these analyses provide initial support for the hypothesis that gF-related differences in activation associated with successful interference control are more likely to be observed in terms of delay-related, rather than probe-related effects.

## Discussion

The results of this study provide evidence for the presence of dual mechanisms of cognitive control over interference in the recent probes task. We have suggested previously [13] that interference may be resolved using two distinct modes of cognitive control: reactive control, which is activated in response to the onset of interference, and proactive control, which is activated prior to the onset of interference. During the recent probes task, we replicated the typical findings within left lateral PFC of greater activation for recent negatives than novel negatives and for recent positives relative to novel positives. This pattern was observed during the post-probe period, consistent with the presence of reactive control mechanisms in left lateral PFC [15]. More importantly, we also found patterns of proactive control activity (i.e., changes in activation during the pre-probe delay period resulting from interference expectancy) in several regions previously implicated in WM.

In addition, the results suggest that proactive and reactive mechanisms of cognitive control are differentially affected by interference expectancy. In several regions previously implicated in WM, there was more evidence of activity related to reactive control in the LE condition than the HE condition, but there was more evidence of activity related to proactive control in the HE condition than the LE condition. We found no evidence of increased delay-period activity in WM regions during the LE condition relative to the HE condition, and in the HE condition only a single region was detected in which probe activity was greater for recent negatives than novel negatives.

This pattern suggests that both proactive and reactive control modes may be successful at controlling interference, but that they may carry different advantages under different task contexts. Namely, it appears that proactive control mechanisms are utilized to a greater extent when greater interference is expected. On the other hand, reactive control mechanisms may operate to resolve interference when it is unexpected, or expected to be infrequent or inconsistent. Although proactive control mechanisms may be more effective at controlling interference, it may not always be efficient to utilize those mechanisms. Because proactive control over interference may entail more sustained and consistent activation (i.e., engaged on every trial and for a longer duration), those mechanisms may require substantially more metabolic resources than reactive control mechanisms [13]. Expending limited metabolic resources for proactive control will be most sensible when the expected benefit is greater, such as when interference expectancy is high. In contrast, when interference expectancy is low, it may simply be less metabolically costly to utilize reactive control mechanisms after the onset of interference, despite the increase in errors and response latencies associated with a reactive control mode.

There are some notable similarities between the DMC model and the two-process model of VLPFC by Badre & Wagner [27]. In the two-process model of VLPFC, mid-IFG subserves a post-retrieval selection process to resolve competition among active representations. In addition, anterior VLPFC performs controlled retrieval of task-relevant information in relationship to task decision criteria. Badre & Wagner suggest that controlled retrieval operates before or during retrieval, and that the selection process operates post-retrieval to resolve conflict. This temporal distinction is consistent with the differentiation between proactive and reactive control mechanisms in the DMC model. Therefore, one might explain our results as showing that proactive control mechanisms promote controlled access to episodic memory (i.e., contextual representations), and reactive control mechanisms perform selection among activated and competing representations.

However, there are apparent inconsistencies between the two-process model of VLPFC and the DMC model. First, it is unclear whether the two-process model of VLPFC would have predicted the engagement of anterior VLPFC prior to the probe period. Specifically, Badre & Wagner [27] state that controlled retrieval is required when there is failure to automatically retrieve goal-relevant knowledge. Therefore, the conditions under which controlled retrieval is engaged seem more reactive (reacting to the failure to automatically retrieve goal-relevant information) than proactive (anticipating demand or motivational incentive for controlled retrieval) in nature. Furthermore, Badre & Wagner [11] discuss the involvement of frontopolar cortex in the recent probes task as monitoring the relationship between target familiarity and the encoding context. This process is more necessary in response to recent probes, for which there are multiple contexts, than novel probes, for which there is only one encoding context. As others have noted [15], the recency status of the probe is only available after the onset of probe, as is the retrieval of multiple contexts in the Badre & Wagner model. As such, it seems likely that the proposed involvement of controlled retrieval processes during the recent probes task is primarily reactive in nature.

An additional distinction arises in that the nature of the DMC model is more inclusive than the two-process model of VLPFC, both in terms of potential processes and brain regions that subserve those processes. Stated differently, proactive control mechanisms are not limited to controlled retrieval, and controlled retrieval is not limited to proactive control over interference. Under the DMC model, the controlled retrieval process, operationalized as a biased competition model, might bias the retrieval of task-relevant information either proactively or reactively. Similarly, we have argued that biased competition mechanisms such as the one postulated for selection in two-process model of VLPFC [17], [18] could be activated proactively to promote the processing and selection of task-relevant representations.

A related question about the neural mechanisms of proactive and reactive control is whether certain brain networks can promote both types of control mode by changing their temporal profiles of activation. Previous research [14] found four lateral PFC regions that switched dynamically between control modes following changes in incentive conditions and training, suggesting that the neural mechanisms of cognitive control can be flexibly shifted between proactive and reactive modes. In the current study, there was evidence that pre-SMA showed reactive control during the LE condition and proactive control during the HE condition. However, no lateral PFC regions showed patterns indicative of both proactive and reactive control. It is difficult to know whether the failure to identify lateral PFC regions showing both control modes reflects a functional differentiation between proactive and reactive control modes, a lack of statistical power, or the omission of factors that are critical for dynamic shifts in control within specific brain regions. Numerous factors varied between these studies (e.g., task demands, strategy training, incentives), and additional investigation of these factors might elucidate the conditions necessary for flexible shifts between control modes. Therefore, further studies will be necessary in order to determine whether proactive and reactive modes of interference control recruit similar brain networks during the recent negative task.

One notable exception to the separation between proactive and reactive control networks in the current study was the dual nature of activation dynamics observed in ACC. Namely, the patterns of activation in dACC/pre-SMA suggested that this region may exert control reactively (i.e., after the onset of recent negative probes) as well as proactively (i.e., prior to the probe period during HE condition). This pattern is in support of previous studies that have suggested the role of dACC in both anticipatory control and conflict-related control processes [29], [30]. The DMC model [13] postulates that ACC plays a role in both proactive and reactive control networks, because it serves to integrate conflict over a short time-scale to signal the immediate need for reactive control [31], and it may also integrate repeated interference over a longer time-scale to signal the need for proactive control in anticipation of conflict [32].

The current study provided initial evidence of differences in the manner by which high gF and low gF groups engaged neural mechanisms of interference control [10]. Compared to the low gF group, the high gF group demonstrated not only better overall accuracy, but also reduced effects of interference on accuracy. This behavioral pattern was paralleled by a tendency for increased delay-related activity in PFC in the high gF group, but increased probe activity associated with recent negatives in the low gF group. These activation patterns suggest that the high gF group activated proactive control mechanisms to a greater degree, and reactive control mechanisms somewhat less, than the low gF group. This increased utilization of proactive control mechanisms may explain the performance benefits demonstrated by the high gF group relative to the low gF group. It nevertheless be noted that these conclusions must be treated as somewhat tentative given low statistical power associated with the small sample size (for between-groups analysis) employed, and relatively circumscribed effects observed.

The present results adjudicate between classes of models regarding how control mechanisms protect the contents of WM from interfering information. Some theories have proposed that interference control mechanisms act to reduce the presence of irrelevant information outside of WM. This reduction may have the consequence of preventing irrelevant information from entering WM, thereby protecting its contents from interference [33],[34]. Other theories suggest that active maintenance of goal-related information in WM biases task-relevant processing, which has the consequence of decreasing the presence of task-irrelevant information and interference caused by it [16], [18], [35].

The current results suggest that the active maintenance of goal representations in WM may be a viable method of preventing interference in a proactive manner. Namely, high interference expectancy is associated with increased activation of lateral PFC regions prior to the probe period, along with a reduction in the number of lateral PFC regions that show differential activation in response to recent negative probes. Importantly, this shift toward proactive control and away from reactive control is coupled with equivalently high performance despite an increased amount of interfering information present in the task. Proactive activity in lateral PFC may reflect the preparation and maintenance of task-goal representations, to facilitate the optimal updating and integration of memory-set information into an attentional bias regarding the upcoming probe. The suggestion that activated representations in lateral PFC may bias competition and reduce interference susceptibility during the recent negatives task is not a new one [36], [37]. However, unlike previous accounts, our results suggest that biased competition may also be beneficial as a proactive control mechanism for preventing interference, as demonstrated by the improved performance by individuals with high gF compared to those with low gF.

It is important to note that other studies have demonstrated a relationship between increased activation for recent negative probes and reduced behavioral interference effects (e.g., [11], [12]). This pattern indicates that interference control may operate through the increased recruitment of reactive mechanisms. These previous findings are not necessarily contradictory with the current results. Indeed, it is important to note that several lateral PFC regions demonstrated increased activation following recent negative probes during the LE condition, but there was a shift in lateral PFC toward more proactive mechanisms during the HE condition. This pattern suggests that differences between studies could result from myriad experimental and individual difference factors that vary incidentally between different studies of interference control.

One factor that could affect the mechanisms of interference control utilized by individuals is their level of gF. Previous studies from our laboratory [5] have demonstrated that higher gF and WM span are related to reduced behavioral interference during the n-back task, but those task designs did not allow investigation of whether the reduced interference susceptibility resulted from proactive or reactive control mechanisms. The results of the current study suggest that individual differences in gF are more likely related to proactive control mechanisms, in that the high gF group demonstrates increased activation of right lateral PFC regions prior to the probe compared to the low gF group. Furthermore, despite having reduced behavioral interference, the high gF group also shows evidence for reduced recruitment of reactive control mechanisms, similar to the pattern observed elsewhere with high WM span individuals [10]. From these results, it seems possible that individuals with high gF more easily engage proactive control mechanisms to curtail interference compared to individuals with low gF, resulting in improved performance. However, the data indicate that individuals with low gF are capable of recruiting proactive control mechanisms under more demanding situations (e.g., high interference expectancy), albeit to a lesser extent than individuals with high gF. It seems feasible that the reduced efficiency with which low gF individuals recruit proactive control mechanisms explains their increased dependence upon reactive control mechanisms regardless of the expected level of demand for interference control.
